# High Expression of FCRLB Predicts Poor Prognosis in Patients With Colorectal Cancer

**DOI:** 10.3389/fgene.2022.882307

**Published:** 2022-06-16

**Authors:** Xiaopeng Wang, Ruirong Lin, Yi Zeng, Yi Wang, Shenghong Wei, Zhitao Lin, Shu Chen, Zaisheng Ye, Luchuan Chen

**Affiliations:** Department of Gastrointestinal Surgical Oncology, Fujian Medical University Cancer Hospital, Fujian Cancer Hospital, Fuzhou, China

**Keywords:** FCRLB, colorectal cancer, biomarker, prognosis, TCGA

## Abstract

**Background:** Mining the prognostic biomarkers of colorectal cancer (CRC) has important clinical and scientific significance. The role of Fc receptor-like B (FCRLB) in solid tumors has never been reported or studied to our knowledge, and the prognostic role of FCRLB in CRC still awaits characterization.

**Methods:** The potential prognostic factor FCRLB was screened out through TCGA database analysis. Then, its expression and associations with clinicopathological variables were assessed in the TCGA CRC cohort. The prognostic value of FCRLB was examined with multiple methods, such as the Kaplan-Meier method, ROC curve, time-dependent ROC analysis, and prediction model nomograms. Then, functional enrichment and annotation among the high and low FCRLB groups were achieved utilizing GO and KEGG analyses and GSEA. Fresh CRC tissue samples obtained clinically were used for the preparation of the tissue microarray and for further validation.

**Results:** FCRLB was highly expressed in CRC tissues compared to normal tissues. Moreover, over-expression of FCRLB correlated with higher CEA levels, advanced T stage, N stage, M stage, AJCC stage, lymphatic invasion, perineural invasion, and incomplete resection (R1 and R2 resection). In addition, high expression of FCRLB was closely correlated to less favorable OS, DSS, and PFI. The analysis of CRC tissue microarray further confirmed the conclusion drawn from the TCGA data analysis.

**Conclusion:** FCRLB is notably up-regulated in CRC tissues and may serve as a potential biomarker of CRC.

## Introduction

Colorectal cancer (CRC) is the third most common type of malignant tumor and the second most frequent leading cause of cancer-related deaths worldwide (Daniel et al., 2017). The mortality rate among patients with CRC has gradually decreased in the past decades, mainly owing to advances in medical technology and treatment. Nevertheless, the prognosis for patients with advanced CRC remains extremely poor, with the 5-year survival rate being less than 14% (Siegel et al., 2018). Thus, determining effective prognostic and predictive markers for early detection and diagnosis of CRC is of great scientific interest and clinical importance.

In recent years, the tumor microenvironment (TME) has gained increasing attention, largely because of its close association with the effectiveness and sensitivity of immunotherapy. Currently, immunotherapy has revolutionized cancer therapy and has rapidly become the mainstay of treatment for a variety of solid cancers, such as malignant melanoma (Komenaka et al., 2004), non-small-cell lung cancer (Nasser et al., 2020), renal cell carcinoma (Deleuze et al., 2020), etc. However, CRC immunotherapy presents enormous challenges to personalized treatment regimens, mainly due to the heterogeneity and dynamics of TME (Ganesh et al., 2019). Tumor-infiltrating immune cells (TIICs) in TME consist of a variety of immune cells, frequently dominated by B cells, as well as other non-tumor cells including T cells, NK cells, and myeloid-lineage cells (e.g., macrophages, mast cells, and neutrophils, etc.). Accumulating evidence indicates that the types and constituents of TIICs in TME not only affect the proliferative and metastatic ability of cancer cells but also affect tumor responses to immunotherapy. For example, it has been reported that tumor-associated macrophages (TAMs) often display characteristics similar to M2 macrophages, which can exert multiple cancer-promoting functions in TME (Cao et al., 2018). Cancer-associated fibroblasts (CAFs), one of the important cellular components of the TME, have been proven to play critical roles in cancer cell proliferation, tumor immunity, extracellular matrix remodeling, and inflammatory responses by secreting cell growth factors, inflammatory factors, and extracellular matrix (Kobayashi et al., 2019). Moreover, clinically, the exhaustion of T cells, the predominant type of immune cell, characterized by the gradual functional deficits of exhausted T cells, has been considered as a cause of the failure of immunotherapy (e.g., chimeric antigen receptor T cell immunotherapy [CAR-T] and immune checkpoint inhibitors) Terranova-Barberio et al., 2020. Accordingly, it is crucial to conduct a comprehensive analysis of tumor-infiltrating immune cell components in TME.

In the present study, we perform bioinformatic analysis of the ribonucleic acid (RNA)-sequencing dataset obtained from The Cancer Genome Atlas (TCGA) project and the Gene Expression Omnibus (GEO) database. Fc receptor-like B (FCRLB) was then screened as a potential prognostic factor for CRC. Furthermore, multifaceted analyses of FCRLB through the TCGA database, such as differential expression, survival analysis, immune infiltration, and potentially related biological pathways, were performed to clarify the clinical utility and prognostic value of FCRLB in CRC and investigate its potential biological mechanisms. Moreover, we use clinically obtained specimens and tissue microarrays (TMA) for further verification of the conclusion drawn from the analysis of the TCGA CRC cohort.

## Methods

### Experimental Design and Data Acquisition

**FIGURE 1:**
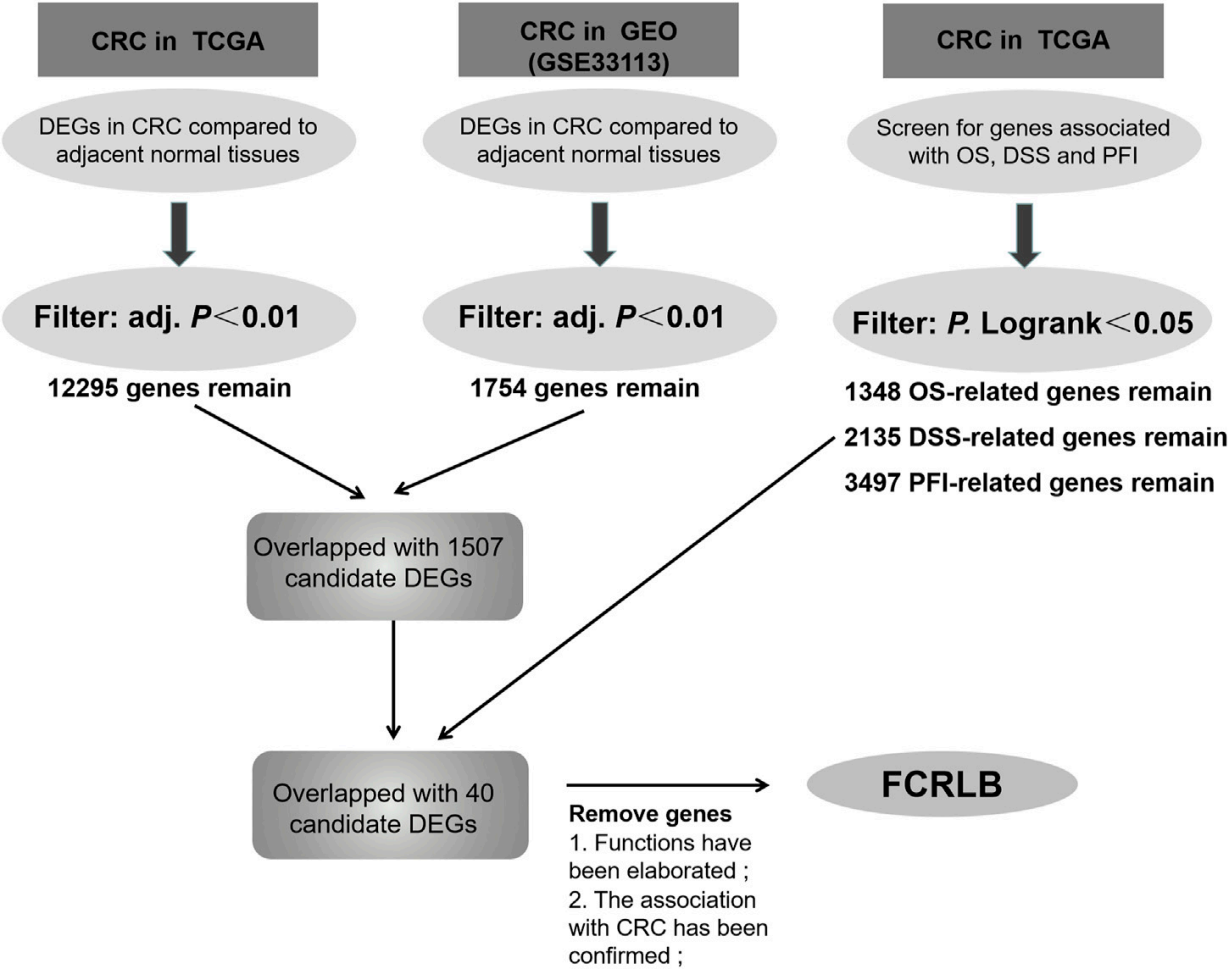
The screening flow chart for selected DEGs associated with prognosis of CRC patients.

The specific experimental process was shown in the flowchart (Figure 1). Level 3 RNA-seq V2 data sets and matched clinicopathological information were downloaded from the TCGA CRC cohort (https://portal.gdc.cancer.gov/; 15 December 2018). Gene expression data of GEO GSE331133 was downloaded. Then the transcriptome data were normalized by using the DESeq package as previous literature described. The limma R package and survival R package were used for screening differentially expressed genes (DEGs) and prognosis-correlated genes, respectively.

### Patients Specimens and Tissue Microarray

Fresh cancerous tissues and matched adjacent non-tumorous tissues were obtained from each CRC patient who underwent surgery in Fujian Cancer Hospital. The normal colorectal tissue, at a distance of 2 cm from the tumor tissue, was excised as the paracancerous tissue specimen. Then, fresh cancerous tissues and matched adjacent non-tumorous tissues were confirmed by histopathological examination, and then were fixed using 10% formalin overnight, and then embedded in paraffin. Tissue samples were prepared in a tissue microarray (TMA), which contained 40 pairs of CRC tissues and matched adjacent normal tissues, as previously described (Xu et al., 2021). TMA was then used for histopathological validation by performing immunohistochemistry (IHC).

### Gene Set Enrichment Analysis

GSEA of microarray data was carried out by using the GSEA v.30 desktop software (Broad Institute) to explore the potential biological mechanisms of FCRLB. If they reached the preset threshold of *p* < 0.05, the biological processes were considered to be apparently enriched.

### GO and KEGG Enrichment Analyses

R package was used for GO and KEGG enrichment analyses and visualization as previously described (Yu et al., 2012). ClusterProfiler R package was used for enrichment analysis, Org. Hs. eg.db R package [3.10.0 version] was used for ID conversion, and GO plot R package [1.0.2 version] was used for calculating Zscore.

### Immune Cell Infiltration in CRC

GSVA R package (version 1.34.0) was used to explore the enrichment of immune cells in the FCRLB high expression and low expression groups as previously described (Hänzelmann et al., 2013). Tumor purity analysis was performed by using Estimate R package according to a previous study (Aran et al., 2015). All procedures were performed using R software (version 4.1.3).

### Immunohistochemistry

The TMA was used to perform immunohistochemistry according to the methods described in the previous literature (Xu et al., 2021). The expression of FCRLB was detected with a special antibody dilution (proteinech, China, 1:100). Then the stained TMA was examined by two expert pathologists independently, who were both blinded to the detailed clinico-pathological data. The number of positive cells in five high-power fields was randomly counted and then protein expression of FCRLB was determined by evaluating the staining intensity of positive staining, scored as negative (less than 25%), weak (25%–50%), medium (50%–75%), and strong (>75%).

### Statistical Analysis

Analysis was performed by using R (version 4.1.3). The data in figures and tables were presented as mean ± SD. The Kaplan-Meier method and the Log-rank test were used to determine the overall survival (OS), disease specific survival (DSS), and progress free interval (PFI) between different groups. Spearman’s correlation coefficient was utilized to analyze the correlation between FCRLB expression and the promoter methylation level. Logistic regression analysis was performed to detect the correlation between FCRLB expression and clinico-pathological parameters. Fisher’s exact tests were used to analyze the correlation between FCRLB expression in CRC tissues and that in paired non-tumorous tissues. Student’s two-tailed unpaired t-test was performed to analyze the FCRLB expression in different groups. Differences at *p* < 0.05 were considered to be significantly different.

## Results

### FCRLB Was Screened as a Differentially Expressed Prognosis-Related Gene

**FIGURE 2:**
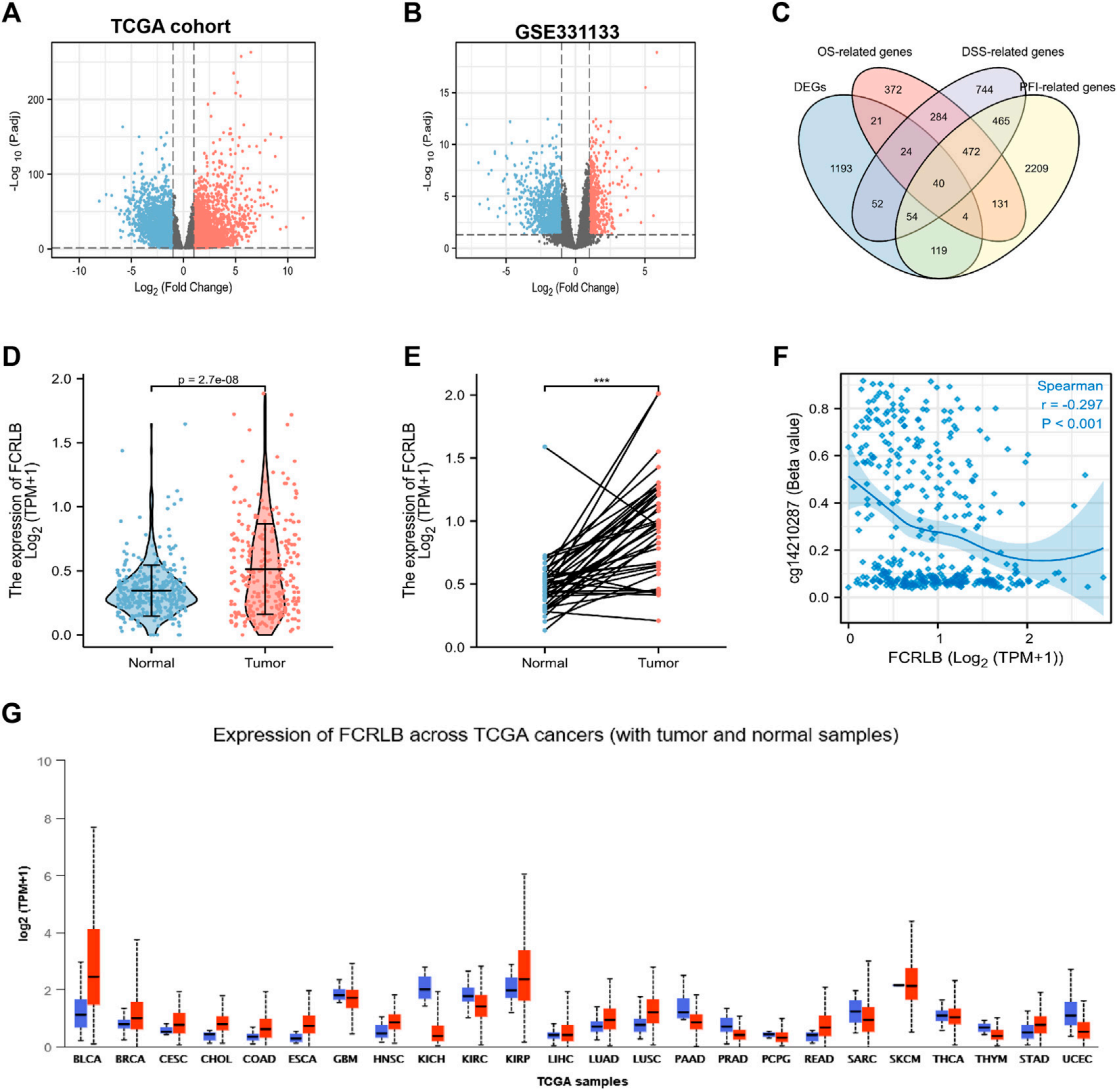
FCRLB was highly expressed in CRC tissues and a variety of tumors. **(A)** The volcano plot showed the DEGs identified between the CRC and normal groups in TCGA cohort. **(B)** The volcano plot showed DEGs identified between CRC and normal groups in GEO database. The red and blue dot represents the up-regulated gene and down-regulated gene with significance, respectively. **(C)** Venn diagram showing genes screened by different methods. **(D)** Expression differences of FCRLB between 647 cancerous tissues and 51 non-tumorous tissues in the TCGA cohort. **(E)** Expression differences of FCRLB between 50 cancerous tissues and 50 matched adjacent normal tissues in the TCGA cohort. ***p < 0.001. **(F)** FCRLB expression was negatively correlated with promoter methylation (r = −0.297, *p* < 0.001). **(E)** FCRLB was overexpressed in a variety of tumors in the TCGA cohort. **(G)** Expression level of FCRLB in pan-cancer.

Data were obtained from the TCGA database and GEO database, and microarray analyses were then performed using the Limma R package to compare the gene expression profiles of CRC and normal tissue groups. A total of 12,295 up-regulated genes were detected in the TCGA database (Figure 2A) and 1,754 in the GEO database (GSE331133) (Figure 2B), based on the screening parameters set at a probability value of *p* < 0.01. The survival R package was used for screening of OS-related genes, DSS-related genes, and PFI-related genes based on the parameters set with *P*. Logrank <0.05. Then 1,348, 2,135, and 3,497 genes that can affect OS, DSS, and PFI were detected, respectively. To further study the DEGs associated with different prognoses, these high-throughput analysis data overlapped, and only 40 candidate genes were left (Figure 2C). Detailed information of candidate genes was upload to the Supplementary Materials. Then genes, whose functions have been elaborated previously or whose association with CRC has been confirmed in a previous study, were eventually abandoned. FCRLB was finally found. A schematic of the screening process of DEGs is shown in Figure 1.

### Expression of FCRLB Was Remarkably Overexpressed in CRC and Multiple Types of Cancers

The analysis of TCGA CRC data revealed that FCRLB was notably up-regulated in CRC tissues as compared to normal tissues (*p* < 0.001) (Figure 2D). Furthermore, FCRLB was highly expressed in CRC tissues compared to matched adjacent normal tissues in the TCGA CRC cohort (*p* < 0.001) (Figure 2E). Aberrant DNA methylation (including hyper- and hypo-methylation) at promoter regions has been reported to be closely associated with changes in gene expression. Our results showed that the expression of FCRLB was significantly negatively correlated with its promoter methylation levels (Spearman correlation = -0.297, *p* < 0.001) (Figure 2F). In addition, a pan-cancer analysis demonstrated that FCRLB was up-regulated in a variety of cancers, such as bladder urothelial carcinoma (BLCA), breast invasive carcinoma (BRCA), cervical squamous cell carcinoma (CESC), cholangiocarcinoma (CHOL), esophageal carcinoma (ESCA), head and neck squamous cell carcinoma (HNSC), lung adenocarcinoma (LUAD), lung squamous cell carcinoma (LUSC), and stomach adenocarcinoma (STAD), as shown in Figure 2G.

### Association Between FCRLB Expression and Clinicopathological Characteristics

**FIGURE 3:**
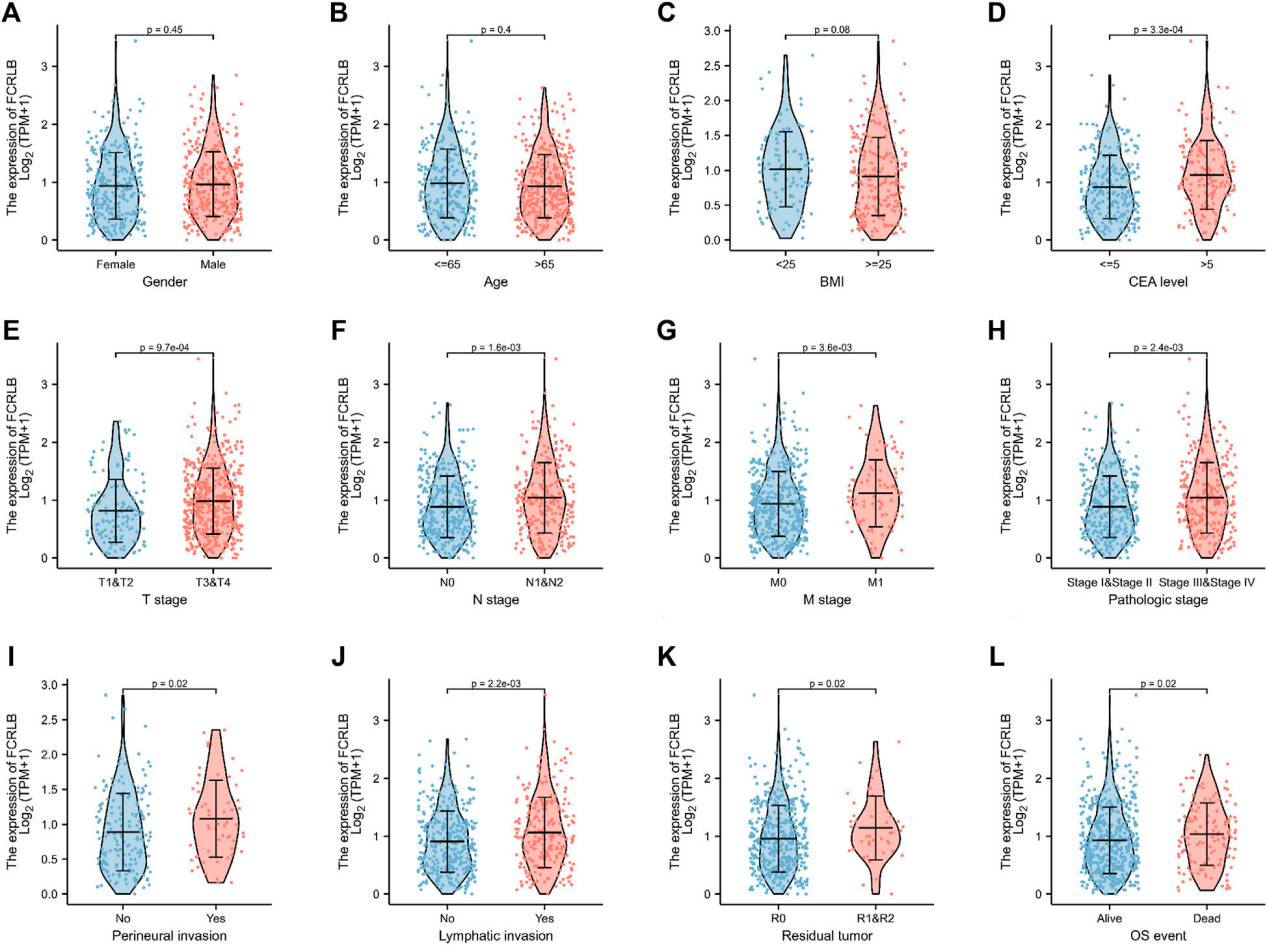
The relationship between FCRLB expression and clinicopathological characteristics in the TCGA CRC cohort. **(A–I)** The FCRLB was highly expressed in CRC groups with higher serum CEA level **(D)**, higher T stage **(E)**, higher N stage group **(F)**, higher N stage group **(G)**, more advanced AJCC stage **(H)**, perineural invasion **(I)**, lymphatic invasion **(J)**, R1/R2 resection **(K)**, more unfavorable OS event **(L)**. However, no significant difference in expression of FCRLB was detected in different gender groups **(A)**, different age groups **(B)** and different BMI groups **(C)**. *p*-values were noted in each figure.

Clinicopathological data obtained from the TCGA CRC cohort, including gender, age, BMI, serum carcinoembryonic antigen (CEA) level, perineural invasion, lymphatic invasion, residual tumor, TNN classification, and OS event, were analyzed. As shown in Figures 3A–C, the FCRLB expression level showed no significant differences between different gender subgroups (Figure 3A), age subgroups (Figure 3B), and BMI subgroups (Figure 3C). In addition, FCRLB expression levels were significantly higher in the subgroup with a higher serum CEA level (*p* < 0.001) (Figure 3D). The results revealed that the expression levels of FCRLB in the tissues of patients at the N1 and N2 stages were significantly higher than those in patients with N0 stage (*p* < 0.01) (Figure 3F). In addition, the expression levels of FCRLB were significantly up-regulated among patients with distant metastasis (M1 stage) than in those without (M0 stage) (*p* < 0.01) (Figure 3G). Moreover, the FCRLB expression levels were much higher among patients in the advanced AJCC stage compared to the early stage (*p* < 0.01) (Figure 3H). Moreover, FCRLB expression in the CRC tissues with incomplete resection (R1 or R2 resection) was notably higher than that with RO resection (*p* < 0.05) (Figure 3K) . As shown in Figures 3I,J, FCRLB expression was strongly associated with perineural invasion (*p* < 0.05) (Figure 3I) as well as lymphatic invasion (*p* < 0.01) (Figure 3J). Then, logistic regression analysis was performed and it revealed that FCRLB expression level was significantly correlated with CEA level (OR = 2.373 (1.574–3.610), *p* < 0.001), T stage (OR = 1.992 (1.346–2.975), *p* < 0.001), N stage (OR = 1.743 (1.271–2.396), *p* < 0.001, M stage (OR = 2.010 (1.263–3.253), *p* = 0.004), AJCC stage (OR = 1.636 (1.190–2.253), *p* = 0.002), residual tumor (OR = 2.174 (1.125–4.423), *p* = 0.025), perineural invasion (OR = 1.865 (1.033–3.418), *p* = 0.040), and lymphatic invasion (OR = 1.480 (1.061–2.070), *p* = 0.021) (Table 1).

**TABLE 1:** Clinicopathological factors and FCRLB expression in the TCGA CRC cohort.

Characteristics	Total (N)	Odds Ratio (OR)	*p* Value
Age (>65 vs. ≤65)	644	0.837 (0.612–1.144)	0.265
Gender (male vs. female)	644	1.119 (0.821–1.526)	0.477
BMI (≥25 vs. <25)	329	0.689 (0.432–1.095)	0.116
CEA level (>5 vs. ≤5)	415	2.373 (1.574–3.610)	<0.001
T stage (T3 and T4 vs. T1 and T2)	641	1.992 (1.346–2.975)	<0.001
N stage (N1 and N2 vs. N0)	640	1.743 (1.271–2.396)	<0.001
M stage (M1 vs. M0)	564	2.010 (1.263–3.253)	0.004
AJCC stage (Stage III and Stage IV vs. Stage I and Stage II)	623	1.636 (1.190–2.253)	0.002
Residual tumor (R1 and R2 vs. R0)	510	2.174 (1.125–4.423)	0.025
Perineural invasion (yes vs. no)	235	1.865 (1.033–3.418)	0.040
Lymphatic invasion (yes vs. no)	582	1.480 (1.061–2.070)	0.021
Neoplasm type (rectum vs. colon adenocarcinoma)	644	1.176 (0.826–1.677)	0.368

### High Expression of FCRLB Predicted Unfavorable Prognosis in the TCGA CRC Cohort

**FIGURE 4:**
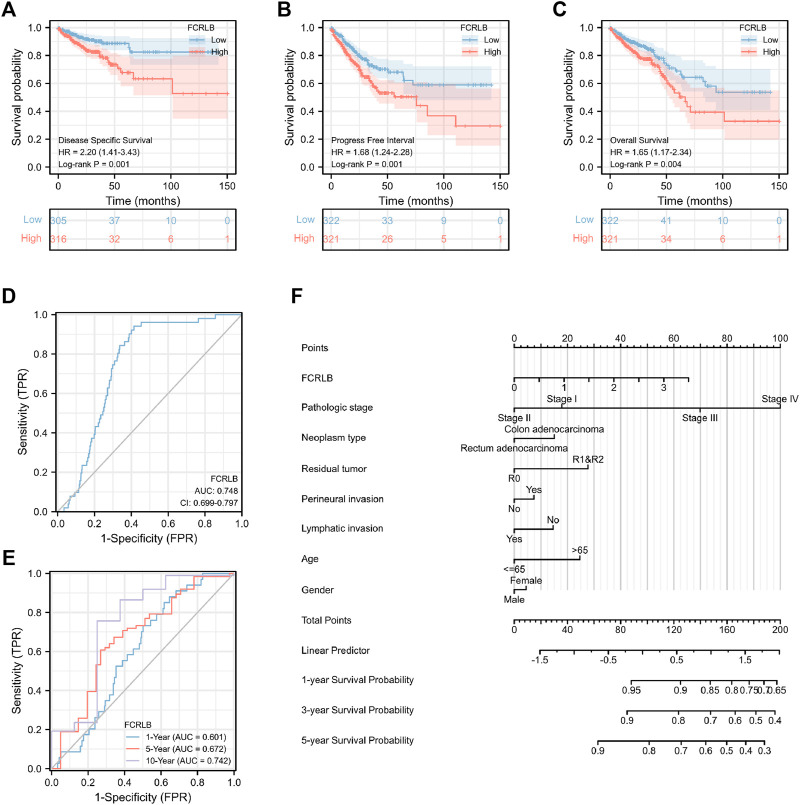
Overexpression of FCRLB was associated with poor prognosis in the TCGA CRC cohort. **(A)** Kaplan-Meier analyses for OS according to FCRLB expression in CRC patients in the TCGA CRC cohort. **(B)** Higher expression of FCRLB was correlated with less favorable DSS in CRC patients in the TCGA CRC cohort. **(C)** Higher FCRLB expression level was correlated with poorer PFI in the TCGA CRC cohort. **(D)** The predictive value of FCRLB was evaluated by ROC curve. **(E)** Time-dependent ROC curves analysis of FCRLB. **(F)** OS Nomogram representation of the multivariate model.

We then analyzed the association between FCRLB expression and the prognosis of CRC patients in TCGA. As shown in Figure 4, low expression of FCRLB exhibited more favorable OS, PFI, and DSS outcomes (*p* < 0.05) (Figures 4A–C). To evaluate the diagnostic value of FCRLB, a receiver operating characteristic (ROC) curve was plotted according to the data obtained from the TCGA cohort. As shown in Figure 4D, the area under the ROC curve was 0.748 [95% confidence interval (CI): 0.699–0.797]. Then, time-dependent ROC curve analysis was constructed to further evaluate the diagnostic accuracy, sensitivity, and specificity of the FCRLB in terms of survival time. In the testing set, the 1-year AUC was 0.601, the 5-year AUC was 0.672, and the 10-year AUC was 0.742 (Figure 4E). In addition, OS nomograms were created to predict the probability of goal attainment based on clinicopathological characteristics (Figure 4F). In sum, the results showed that the overexpression of FCRLB was an unfavorable prognostic factor in CRC patients and presented good predictive performance.

**FIGURE 5:**
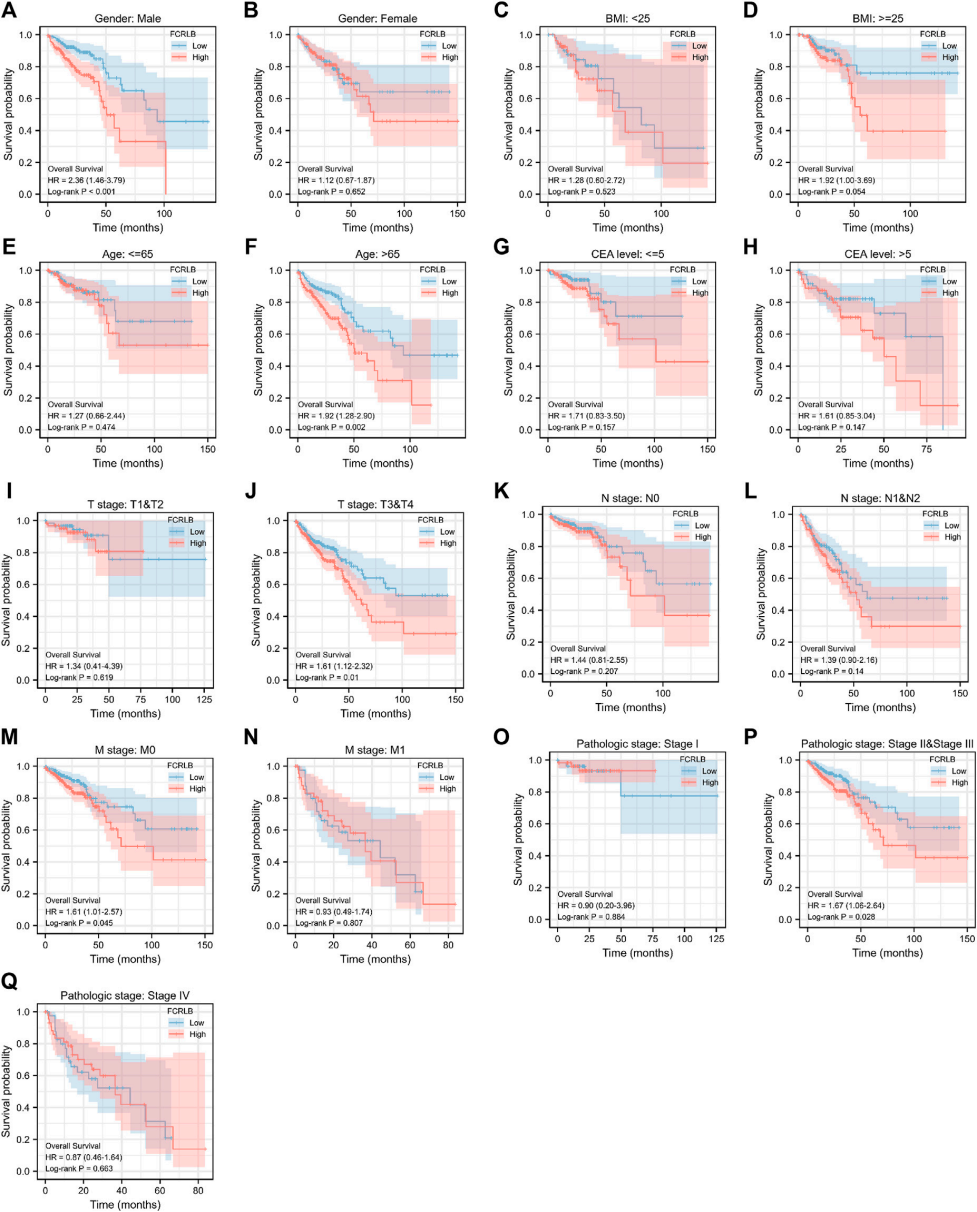
Kaplan-Meier OS curves for FCRLB expression in different clinical subgroups. (A-S) Kaplan-Meier OS curves for FCRLB expression in male subgroup **(A)**, female subgroup **(B)**, BMI < 25 subgroup **(C)**, BMI < 25 subgroup **(D)**, age ≤65 subgroup **(E)**, age < 65 subgroup **(F)**, CEA level ≤5 subgroup **(G)**, CEA level≤ >5 subgroup **(H)**, T1 and T2 stage subgroup **(I)**, T3 and T4 stage subgroup **(J)**, N0 stage subgroup **(K)**, N1 and N2 stage subgroup **(L)**, M0 stage subgroup **(M)**, M1 stage subgroup **(N)**, AJCC pathologic stage I subgroup **(O)**, AJCC pathologic stage II and III subgroup **(P)**, AJCC pathologic stage IV subgroup **(Q)**.

In order to further characterize the role of FCRLB in predicting survival, survival analysis in 17 clinical subgroups was then conducted based on clinic-pathological characteristics such as gender, BMI, age, CEA level, T stage, N stage, M stage, and AJCC pathologic stage. As shown in Figure 5A–Q, FCRLB was identified as an independent prognostic and predictive factor in male patients (Figure 5A), old patients (age > 65) (Figure 5F), T2-3 stage patients (Figure 5J), patients without metastasis (M1 stage) (Figure 5M), and AJCC stage II–III stage patients (Figure 5P).

### FCRLB Was Highly Expressed in CRC Tissues and Correlated With Infiltrating and Metastatic Ability in CRC Tissue Microarray (TMA)

**FIGURE 6:**
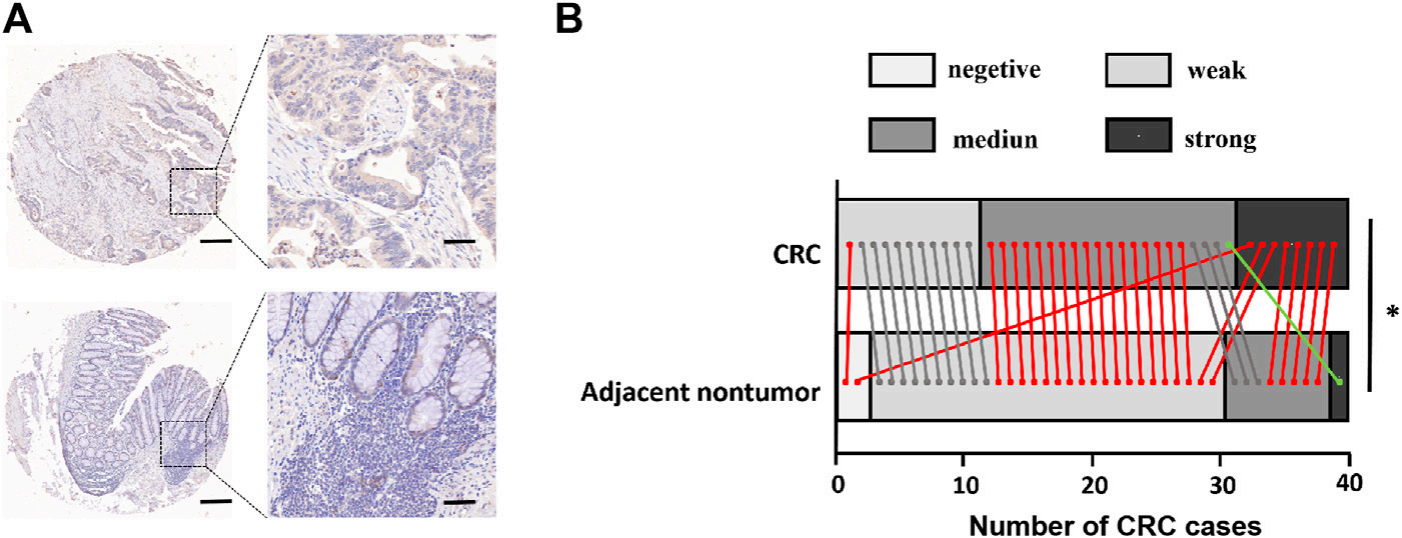
FCRLB protein was highly expressed in CRC. **(A)** Representative image of immunohistochemical staining of FCRLB on a TMA containing 40 pairs of CRC and adjacent normal tissues. Scale bars = 500 μm (right) or 100 μm (left). **(B)** Statistical analysis of IHC staining results for FCRLB from the CRC tissue microarray slide. *p < 0.05.

First, the expression level of FCRLB in CRC tissues and adjacent normal tissues was compared using constructing and staining tissue microarray analysis. Representative pictures are shown in Figure 6A. Moreover, TMA revealed that FCRLB was strongly expressed in 8 of 40, moderately expressed in 20 of 40, and weakly expressed in 11 of 40 CRC tissues, respectively. By contrast, FCRLB was strongly expressed in 1 of 40, moderately expressed in 8 of 40, weakly expressed in 29 of 40, and negatively expressed in 2 of 40 adjacent normal tissues, respectively (*p* < 0.001) (Figure 6B). In addition, we analyzed the correlation between the expression level of FCRLB and the clinicopathological parameters of CRC patients. FCRLB was closely related to invasion depth (*Χ* ^2^ = 5.625, *p* < 0.05) and lymphatic metastasis (*Χ* ^2^ = 3.956, *p* < 0.05)) of CRC (Table 2.). Detailed clinicopathological information of CRC TMA was in Supplementary Materials.

**TABLE 2:** Clinicopathological factors and FCRLB expression in TMA.

Characteristics	Low expression (*n* = 20)	High expression (*n* = 20)	*Χ* ^2^	*p* Value
Age (>65)	10	8	0.404	0.525
Gender (male vs. female)	12	9	0.902	0.304
Differentiation (poorly differentiation)	2	5	1.558	0.212
Tumor size (maximal diameter >3 cm)	13	17	2.133	0.144
Invasion depth (T3 and T4 stage)	13	19	5.625	0.018
Tumor metastasis (M1 stage)	1	2	0.360	0.548
Lymphatic metastasis (yes vs. no)	4	10	3.956	0.047

### Functional Annotation Among the High and Low FCRLB Expression Groups

**FIGURE 7:**
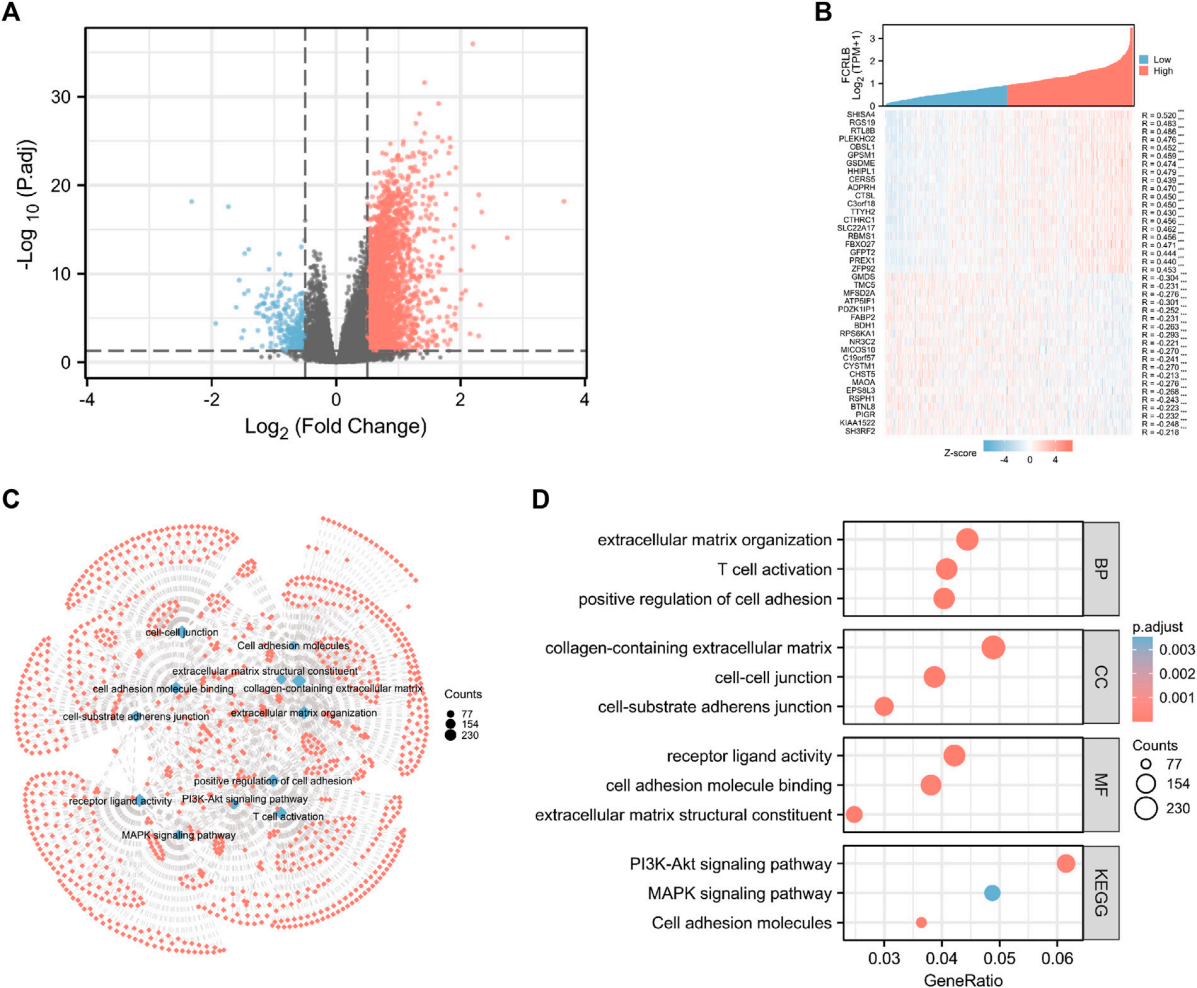
Functional and pathway annotation among the high and low FCRLB groups by GO and KEGG analysis. **(A)** The volcano plot showed the DEGs identified between high and low FCRLB groups in the TCGA CRC cohort. The red and blue dot represents the up-regulated gene and down-regulated gene with significance, respectively, and each grey dot represents genes that did not meet the preset parameters (|log2(FC)|>1 and p. adj<0.05). **(B)** Gene expression heat map and correlations for FCRLB co-expressed genes. *p < 0.05, **p < 0.01**, and *p < 0.001. **(C,D)** The top 12 significant terms of GO and KEGG pathway analyses. The results were presented in a network diagram **(C)** and a bubble chart **(D)**, respectively.

**FIGURE 8:**
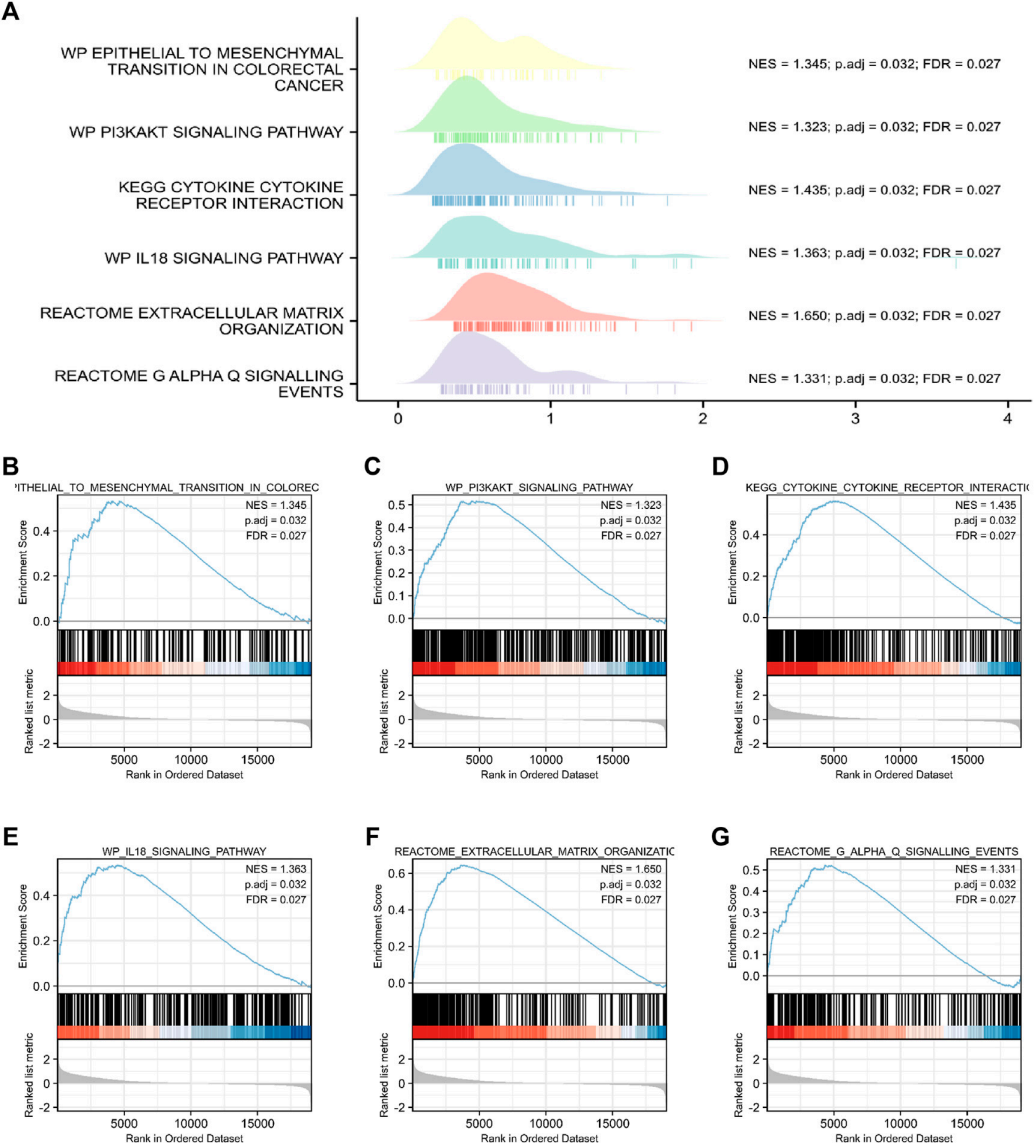
Functional and pathway annotation among the high and low FCRLB groups by GSEA. **(A–G)** The GSEA revealed that EMT **(B)**, PI3K/Akt signaling pathway **(C)**, cytokine and cytokine receptor interaction **(D)**, IL-18 signaling pathway **(E)**, extracellular matrix organization **(F)**, Galphaq signaling **(G)**.

Through the analysis of TCGA data, CRC patients in the TCGA cohort could be divided into FCRLB high and low expression groups according to the FCRLB expression level. Then, differentially expressed genes (DEGs) between the two groups were identified as FCRLB-associated genes. After obtaining the DEGs, through GO/KEGG enrichment analysis or GSEA, it is to further infer the possible functions or pathways involved in FCRLB. The results revealed that 499 DEGs were identified by performing genome-wide co-expression analysis with set cutoff values (*p*.adj <0.05 and |Log2-fold change|>1) between FCRLB high expression group and the low expression group. A total of 458 up-regulated genes and 41 down-regulated genes were identified and presented in the volcano plot (Figure 7A). The top 20 positively correlated genes and the top 20 negatively correlated genes in the TCGA cohort, ordered by the Pearson correlation coefficient, were presented in a gene co-expression heatmap (Figure 7B). The results of GO and KEGG pathway analyses were present in a network diagram and bubble diagram, which showed that co-expression of FCRLB was mainly enriched in extracellular matrix organization, T cell activation, positive regulation of cell adhesion, collagen-containing extracellular matrix, cell-cell junction, cell-substrate adherens junction, receptor ligand activity, cell adhesion molecule binding, extracellular matrix structural constituent, phosphoinositide 3-kinase/AKT (PI3/AKT signaling pathway), mitogen-activated protein kinase (MAPK) signaling pathway, and cell adhesion molecules (Figures 7C,D). In addition, the GSEA was performed to further identify the potential related pathway of FCRLB. The GSEA revealed that FCRLB was enriched in substantial gene sets, including epithelial-to-mesenchymal transition (EMT) in colorectal cancer (NES = 1.345, FDR <0.05) (Figure 8B), PI3/AKT signaling pathway (NES = 1.323, FDR <0.05) (Figure 8C), cytokine and cytokine receptor interaction (NES = 1.323, FDR <0.05) (Figure 8D), interleukin-18 (IL-18) signaling pathway (NES = 1.363, FDR <0.05) (Figure 8E), extracellular matrix organization (NES = 1.650, FDR <0.05) (Figure 8F), and Galphaq signal (NES = 1.331, FDR <0.05) (Figure 8G).

### Correlation Between FCRLB and Tumor Microenvironment in CRC

The tumor microenvironment (TME), including cellular and non-cellular components, is increasingly being recognized to play a critical role in tumorigenesis. According to the previous literature (Rostamzadeh et al., 2018), FCRL family members play a critical role in cell-mediated immunity and tumor immunology. However, the role of FCRLB in modulating TME and tumor immunology has never been addressed before.

**FIGURE 9:**
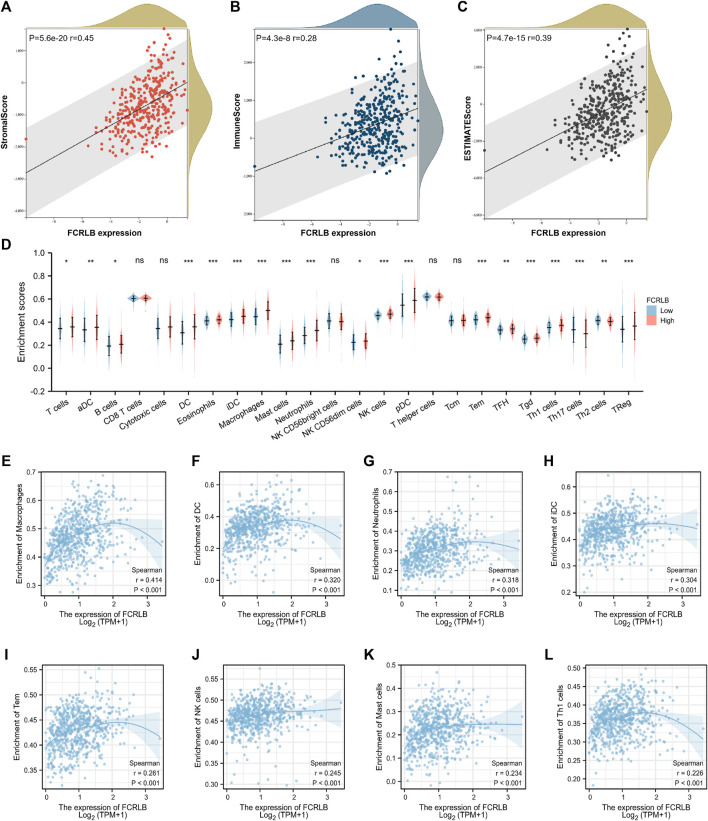
The relationship between FCRLB expression and immune cell infiltration in TCGA CRC cohort. **(A–C)** The relationship between FCRLB expression levels and the StromalScore, ImmuneScore, and ESTIMATEScore. The correlation analysis revealed that FCRLB was positively correlated with the StromalScore **(A)**, ImmuneScore **(B)**, and ESTIMATEScore **(C)**. **(D)** The relationship between FCRLB expression levels and the abundance of different types of immune cells. ns stands for not statistically different. *P < 0.05, **P < 0.01** and *P < 0.001. **(E–L)** FCRLB expression was positively correlated with macrophage infiltration **(E)**, DC infiltration **(F)**, Neutrophil infiltration **(G)**, iDC infiltration **(H)**, Tem infiltration **(I)**, NK cell infiltration **(J)**, Mast cell infiltration **(K)**, Th1 cell infiltration **(L)**, etc. Sperman’s coefficient **(R)** and p-value were noted in each figure.

In TME, tumor purity, representing the percentage of tumor cells, could be calculated by performing the ESTIMATE method. TumorPurity, StromaScore, and ESTIMATEScore represent the percentage of tumor cells, the percentage of immune cells, and the percentage that merge ImmuneScore and StromaScore, respectively. The association between the ImmuneScore (StromalScore and ESTIMATEScore) and FCRLB expression was assessed as shown in Figure 9A–C FCRLB expression was positively correlated with ImmuneScore (r = 0.45, *p* < 0.001), StromalScore (r = 0.28, *p* < 0.001), and ESTIMATEScore (r = 0.39, *p* < 0.001), which demonstrated that FCRLB played a role in remodeling TME.

In order to characterize the immunological role of FCRLB in TME, we further evaluated the relationship between FCRLB and immune cell infiltration in CRC by analyzing the data set in the TCGA cohort and performing a single-sample gene set enrichment analysis (ssGSEA) according to the previous literature (Bindea et al., 2013). The difference in the abundance of immune/stromal cells between the high FCRLB group and the low FCRLB group was shown in Figure 9D. Moreover, the relationship between FCRLB expression and immune cell infiltration was intuitively represented in a bubble diagram (Supplementary Figure S1). The analysis revealed that FCRLB expression was positively correlated with the abundance of multiple immune cells, such as macrophages (r = 0.414, *p* < 0.001) (Figure 9E), dendritic cells (DC) (r = 0.320, *p* < 0.001) (Figure 9F), neutrophils (r = 0.318, *p* < 0.001) (Figure 9G), immature dendritic cells (iDC) (r = 0.304, *p* < 0.001) (Figure 9H), effective memory T cells (Tem) (r = 0.261, *p* < 0.001) (Figure 9I), NK cells (r = 0.245, *p* < 0.001) (Figure 9J), mast cells (r = 0.234, *p* < 0.001) (Figure 9K), and Th1 cells (r = 0.226, *p* < 0.001) (Fig. 9L).

### Correlation Between FCRLB and Immune-Related Modulators in CRC

To better characterize the potential role of FCRLB in modulating TME and tumor immunity, the relationship between FCRLB and immune modulators was further explored by analyzing the association between FCRLB expression and the maker genes of related immune cells.

**FIGURE 10:**
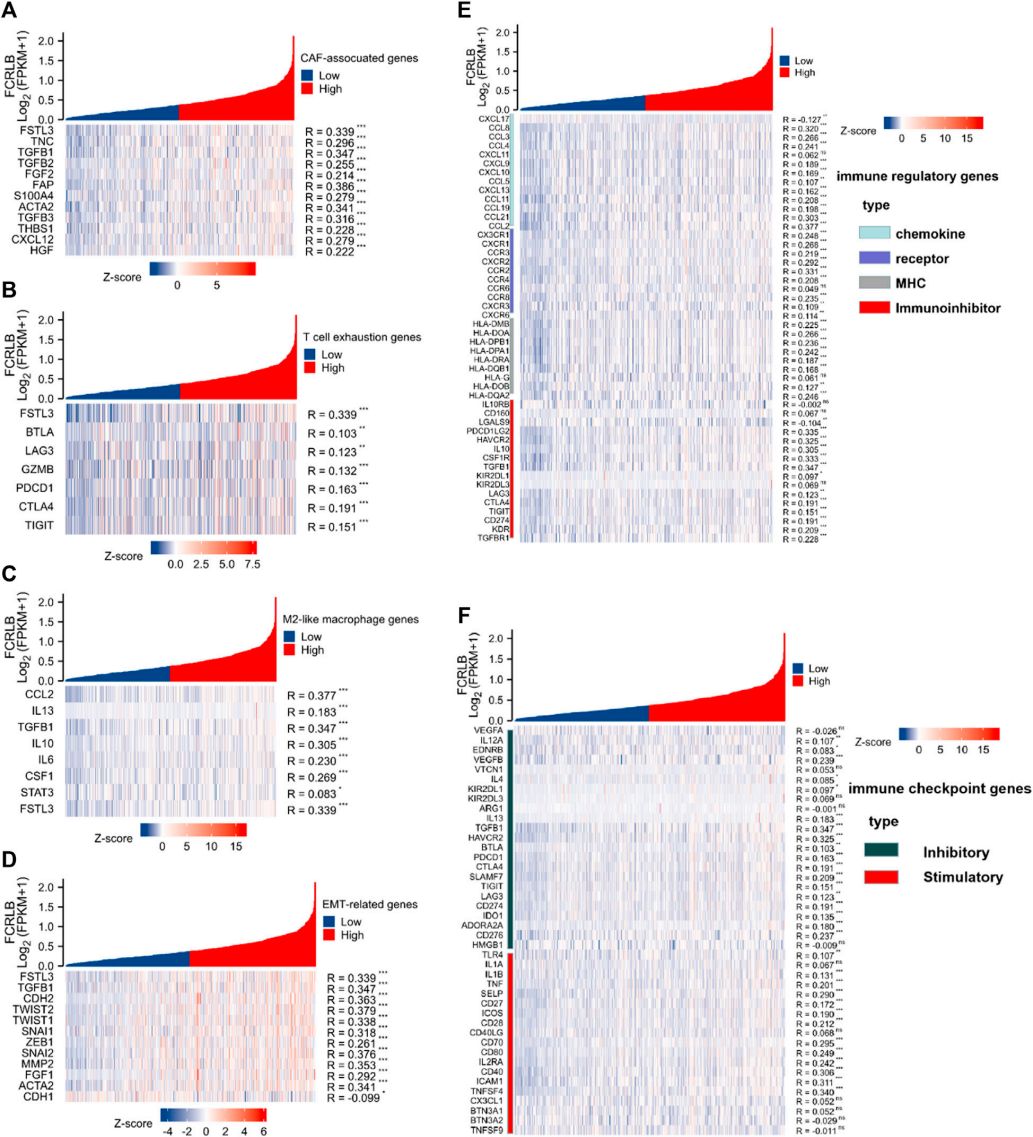
The relationship of FCRLB expression to immune-related modulators and immune checkpoint molecules in the TCGA CRC cohort. **(A–D)** FCRLB was significantly positively correlated with most CAF-related genes **(A)**, T cell exhaustion genes **(B)**, M2 like macrophage genes **(C)**, EMT-related genes **(D)**. **(E)** FCRLB expression level was significantly correlated with majority of the immune regulatory genes. **(F)** FCRLB expression level is notably correlated with the expression of immune checkpoint molecules in CRC. *p < 0.05, **p < 0.01, ***p < 0.001, ns: no significant difference.

As shown in Figure 10A, significant positive correlations between FCRLB expression and the majority of genes related to CAF-associated genes were clearly observed. Moreover, we found that FCRLB expression was positively correlated with the gene set associated with T cell exhaustion (Figure 10B). In addition, FCRLB also showed a significant positive correlation with M2-like macrophage genes (Figure 10C), as well as genes associated with epithelial-to-mesenchymal transition (EMT), which is a well-documented transdifferentiation program playing a critical role in cancer metastasis (Ievgenia and Cédric, 2019) and chemoresistance (Zheng et al., 2015) (Figure 10D).

Not only that, we also explored the relationship between FCRLB expression and immune regulatory genes (including chemokine, receptor, major histocompatibility complex (MHC), and immunoinhibitor). Notably, FCRLB showed a significant positive correlation with the majority of immune regulatory genes.

Immune checkpoint receptors inhibit the activation of T cells by delivering co-inhibitory signals to modulate the duration and intensity of the immune response (Darvin et al., 2018). In our study, the relationship between FCRLB expression and a variety of immune checkpoint molecules in CRC was explored. The results revealed a strong correlation between FCRLB expression and the majority of immune checkpoint molecules in CRC, including inhibitory and stimulatory genes (Figure 10F).

## Discussion

Although the prognosis of patients with CRC has dramatically improved over the past decades, early accurate diagnosis and therapy are still considered important prognostic factors. Prognostic and predictive biomarkers are considered to benefit early detection, accurate diagnosis, and precise therapy of CRC. Therefore, from a clinical standpoint, it is imperative to excavate prognostic and predictive biomarkers.

In our research, based on data mining of the TCGA database, FCRLB was screened as a potential prognostic biomarker for CRC patients. We discovered that FCRLB was overexpressed in CRC tissues compared to adjacent normal tissues. Moreover, we found that the high expression of FCRLB was significantly correlated with unfavorable OS, DSS, and PFI in CRC and may serve as a potential prognostic biomarker of CRC. Furthermore, ROC curve, time-dependent ROC curve, and OS nomogram were created and demonstrated the utility and good predictive performance of FCRLB in CRC. According to the analysis of clinicopathological data in the TCGA CRC cohort, FCRLB expression was positively correlated with a high serum CEA level, an advanced TNM stage, incomplete tumor resections, and perineural and lymphatic invasion, all of which are typically considered poor prognostic factors. Subsequently, we further detected the expression level of FCRLB and explored the relationship between FCRLB expression and clinicopathological characteristics using the TMA technique. Additionally, we demonstrated that the strong expression of FCRLB was strongly associated with lymph node metastasis and invasion depth, which confirmed, to some extent, the conclusions drawn from our analysis of the TCGA database.

Fc receptor-like (FCRL) molecules, a family of cellular receptors, are mainly expressed in B cells (Ehrhardt et al., 2007). To date, eight different members of the FCRL family have been identified in humans, including FCRL-6, FCRLA, and FCRLB. FCRL1-6 encode type I transmembrane glycoproteins with similar extracellular immunoglobulin (Ig)-like domains and intracellular regions that contain consensus tyrosine-based motifs (viz., an immunoreceptor tyrosine-based activation motif and/or an immunoreceptor tyrosine-based inhibitory motif). However, FCRLA and FCRLB mainly serve as intracellular proteins rather than transmembrane receptors like other members of the FCR family (Davis, 2020). The FCRL family has been proven to play a critical role in phagocytosis, antibody-dependent cell cytotoxicity, immediate hypersensitivity, and transcytosis of immunoglobulins via their ability to bind immunoglobulin constant regions (Chikaev et al., 2005). Recently, a large number of studies on the FCRL family in tumors and tumor immunity have emerged. For instance, anti-FCRL1 immunotoxin E9 (Fv)-PE38, a well-designed immunotoxin targeting cell-surface receptors, displayed remarkably selective cytotoxicity on FCRL1-positive malignancies (Du et al., 2008). Furthermore, FCRL2 expression predicts clinical progression in chronic lymphocytic leukemia (Li et al., 2008). Genetic polymorphisms of FCRL3 were also associated with the risk of head and neck cancer in a Chinese population (Zhang and Sun, 2021). However, to the best of our knowledge, very few studies regarding the immunological function of FCRLB have been reported previously, and its role in oncology and tumor immunology has never been addressed, partially due to it being a relatively newly defined group within the FCRL family. In this study, to the best of our knowledge we characterized, for the first time, the potential oncological role of FCRLB.

In addition, to further elucidate the underlying biological mechanism of FCRLB in CRC, GSEA, GO, and KEGG analysis were performed. The results demonstrated that some well-documented tumor-related pathways and immune-related pathways in the FCRLB high expression group were significantly enriched, such as EMT programs, the PI3/AKT pathway, cytokine and cytokine receptor interactions, the IL-18 signaling pathway, extracellular matrix organizations, Galphaq signals, and so on. Accordingly, we speculated that FCRLB may serve as an oncogene that influences the prognosis of patients with CRC through these common biological pathways (Li et al., 2014; Nakamura et al., 2018; Ievgenia and Cédric, 2019; Patra et al., 2021).

In recent years, the tumor immune microenvironment has become an area of intense research interest, mainly due to its close association with the effectiveness of immunotherapy. Most members of the FCRL family ubiquitously serve as transmembrane glycoproteins responsible for the recognition of extracellular ligands by the cellular effector pathways of immune cells. Considering the critical role of the FCRL family in immunoregulation, we explored the potential association between FCRLB and the TME of CRC. GSEA, GO, and KEGG analysis revealed that immune-related pathways were significantly enriched in the FCRLB high expression group, such as T cell activation, cytokine and cytokine receptor interaction, and IL-18 signaling pathways. Moreover, FCRLB expression was significantly positively correlated with the majority of the genes of CAF and M2-like macrophages, which are both immunosuppressive cells. Not only that, a significantly positive correlation was observed between most immunosuppression-associated genes and FCRLB expression. Clinically, T cell exhaustion, characterized by the gradual functional deficits of exhausted T cells, has been considered to be a cause of the failure of immunotherapy. In our research, FCRLB expression correlated positively with T cell exhaustion genes. The immunosuppressive TME is a major barrier to immunotherapy leading to a limited immunotherapy effect (Devalaraja et al., 2020). Thus, we speculated that high FCRLB may lead to the formation of an immunosuppressive TME that can promote immune escape and foster tumor growth. Targeted inhibition of FCRLB may reverse the immunosuppression of TME to improve outcomes for CRC patients. In sum, our study indicated that FCRLB might play an important role in tumor immunity by modulating TME. However, the regulatory mechanism of FCRLB in tumor immunity still needs to be further clarified.

It is undeniable that the current study has some limitations. First, the functional roles of FCRLB in regulating tumorigenesis and the development of colorectal cancer were unclear and need to be addressed with further investigation. Second, the clinical conclusions drawn from the current study were mainly derived from the analysis of public database. Thus, further clinical investigation and validation are urgently needed. Third, in our research, the number of TMA cases was quite small, and the sample size derived from public databases is also limited, which may lead to inaccurate conclusions. Therefore, large samples from multiple datasets are needed to support these conclusions. Moreover, in the data we obtained from the TCGA database, most clinical information about preoperative (neo-adjuvant) or postoperative (adjuvant) therapy was unknown, even as they serve as critical factors affecting prognosis. Therefore, the association between FCRLB expression level and neoadjuvant or adjuvant therapy was not elaborated.

## Conclusion

In conclusion, we confirmed that FCRLB overexpression is closely related to the poor prognosis of patients with CRC. Moreover, we identified FCRLB as a biomarker for evaluating the prognosis of CRC patients. In light of these findings, further studies on the specific function and potential mechanism of FCRLB are urgently warranted, and the development of an efficient strategy to suppress FCRLB expression may improve the prognosis of patients with CRC in the future.

## Data Availability

The datasets presented in this study can be found in online repositories. The names of the repository/repositories and accession number(s) can be found in the article/Supplementary Material.

## Abbreviations

FCRL, Fc receptor like; FCRLB, Fc receptor like B; CRC, colorectal cancer; TCGA, The Cancer Genome Atlas; GSEA, gene set enrichment analysis; GO, gene ontology; KEGG, Kyoto Encyclopedia Genes and Genomes; TMA, tissue microarray; IHC, immunohistochemistry; OS, overall survivals, DSS, disease-specific survivals; PFI, progress free interval; CEA, carcinoembryonic antigen; OR, odds ratio; CI, confidence interval; DEGs, differently expressed genes; MAPK, mitogen-activated protein kinases; PI3/AKT, phosphoinositide 3-kinase/AKT; EMT, epithelial-to-mesenchymal transition; FDR, false discovery rate; TME, tumor microenvironment; ssGSEA, single-sample Gene set enrichment analysis; CAFs, cancer-associated fibroblasts; TAMs, tumor-associated macrophages; ROC, receiver operating characteristic; ECM, extracellular matrix; TIICs, tumor-infiltrating immune cells.
