# Supplementary figures and images for: High Expression of FCRLB Predicts Poor Prognosis in Patients With Colorectal Cancer
Source: Front Genet. 2022 Jun 16;13:882307. doi: 10.3389/fgene.2022.882307 (PMC9244534; doi:10.3389/fgene.2022.882307)

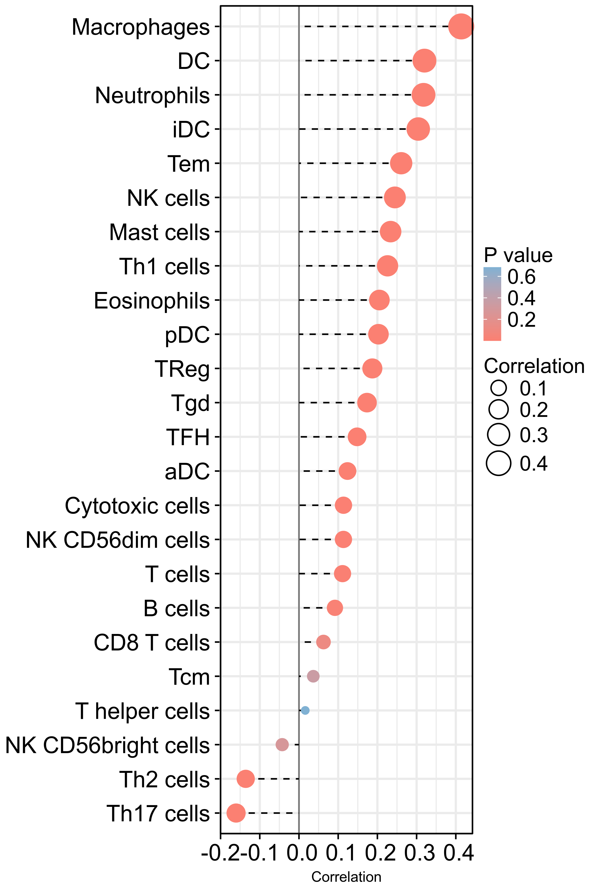

Supplement: Supplementary file 3 [file Image1.TIF]
